# The Wnt signalling pathway is upregulated in an *in vitro* model of acquired tamoxifen resistant breast cancer

**DOI:** 10.1186/1471-2407-13-174

**Published:** 2013-04-02

**Authors:** Yan Ni Loh, Ellen L Hedditch, Laura A Baker, Eve Jary, Robyn L Ward, Caroline E Ford

**Affiliations:** 1 Adult Cancer Program, Level 2, Lowy Cancer Research Centre and Prince of Wales Clinical School, University of New South Wales, New South Wales, 2052, Australia

**Keywords:** Wnt-signalling, Breast cancer, Tamoxifen resistant, Endocrine resistant, Epithelial to mesenchymal transition (EMT), IWP-2

## Abstract

**Background:**

Acquired resistance to Tamoxifen remains a critical problem in breast cancer patient treatment, yet the underlying causes of resistance have not been fully elucidated. Abberations in the Wnt signalling pathway have been linked to many human cancers, including breast cancer, and appear to be associated with more metastatic and aggressive types of cancer. Here, our aim was to investigate if this key pathway was involved in acquired Tamoxifen resistance, and could be targeted therapeutically.

**Methods:**

An *in vitro* model of acquired Tamoxifen resistance (named TamR) was generated by growing the estrogen receptor alpha (ER) positive MCF7 breast cancer cell line in increasing concentrations of Tamoxifen (up to 5 uM). Alterations in the Wnt signalling pathway and epithelial to mesenchymal transition (EMT) in response to Tamoxifen and treatment with the Wnt inhibitor, IWP-2 were measured via quantitative RT-PCR (qPCR) and TOP/FOP Wnt reporter assays. Resistance to Tamoxifen, and effects of IWP-2 treatment were determined by MTT proliferation assays.

**Results:**

TamR cells exhibited increased Wnt signalling as measured via the TOP/FOP Wnt luciferase reporter assays. Genes associated with both the β-catenin dependent (AXIN2, MYC, CSNK1A1) and independent arms (ROR2, JUN), as well as general Wnt secretion (PORCN) of the Wnt signalling pathway were upregulated in the TamR cells compared to the parental MCF7 cell line. Treatment of the TamR cell line with human recombinant Wnt3a (rWnt3a) further increased the resistance of both MCF7 and TamR cells to the anti-proliferative effects of Tamoxifen treatment. TamR cells demonstrated increased expression of EMT markers (VIM, TWIST1, SNAI2) and decreased CDH1, which may contribute to their resistance to Tamoxifen. Treatment with the Wnt inhibitor, IWP-2 inhibited cell proliferation and markers of EMT.

**Conclusions:**

These data support the role of the Wnt signalling pathway in acquired resistance to Tamoxifen. Further research into the mechanism by which activated Wnt signalling inhibits the effects of Tamoxifen should be undertaken. As a number of small molecules targeting the Wnt pathway are currently in pre-clinical development, combinatorial treatment with endocrine agents and Wnt pathway inhibitors may be a useful therapeutic option in the future for a subset of breast cancer patients.

## Background

Among several advances that have contributed to the decreased mortality from breast cancer observed in the past two decades, the routine use of adjuvant endocrine therapies directed at the estrogen receptor (ER) pathway is a major contributor. Tamoxifen, a selective estrogen receptor modulator (SERM) that blocks mammary estrogen action at its receptor, increases patient survival following a diagnosis of ER positive breast cancer [1-3]. The long-term benefit of Tamoxifen, however, is limited by the development of acquired resistance. A recent meta-analysis of adjuvant Tamoxifen for 5-years revealed a 33% distant tumour relapse rate within 8-years post treatment [4,5]. Endocrine relapse predicts a poor clinical outcome as about 40% of these woman have wide-spread disease at the time of clinical presentation. One pathway which has been identified as of potential importance in acquisition of drug resistance to Tamoxifen, is the Wnt signalling pathway [6].

The Wnt signalling pathway is an important developmental pathway, that is frequently dysregulated in human cancers, including breast cancer [6-10]. Wnt signalling is important for cell migration, invasion, adhesion and survival. Wnt ligands primarily signal via membrane bound Frizzled receptors through a number of different but interconnected signalling pathways, including the Wnt/Ca^2+^, β-catenin and planar-cell polarity pathways [11-13]. In general, the Wnt pathway is divided into the canonical/ β-catenin dependent pathway and the non canonical/ β-catenin independent pathways (including the planar cell polarity pathway and Wnt/Ca^2+^ pathway), though it is now understood that there is significant overlap and cross-talk between the individual pathways.

As a consequence of the frequent involvement of the Wnt signalling pathway in multiple cancers, many attempts have been made to target the pathway therapeutically [14]. In general, these attempts have had somewhat limited success, likely due to the complexity of the Wnt network and the fact that many of these inhibitors were targeted further downstream in the pathway. The Wnt inhbitor IWP-2 was recently identified as a small molecule inhibitor of Porcupine, thus capable of inhibiting secretion and activity of all Wnt ligands and downstream pathways [15].

Wnt signalling has also recently been linked to the process of epithelial to mesenchymal transition (EMT) [16-18]. This is unsurprising due to the Wnt pathways’ well established and defined links to cell polarity, differentiation and cell migration. EMT is a crucial step that cancer cells undergo in order to invade and metastasise [19]. Cells that have undergone an EMT possess similiarities to cancer stem cells in their plasticity, loss of adherence and capacity for migration and invasion. In general, the hallmarks of a cancer cell which has undergone EMT is a loss of E-cadherin expression, and gain of Vimentin [20]. The transcription factors Twist and Snail are frequently upregulated in parallel. Another important similarity is with cancer stem cells, in that cells which have undergone an EMT have been shown to more chemoresistant in a number of different tumour types treated with different cancer therapies [21,22].

In this present study we sought to profile the mRNA expression of key Wnt signalling pathway and EMT associated genes in an *in vitro* model of acquired Tamoxifen resistant breast cancer (TamR). The TamR cell line was developed to simulate the occurrence of acquired Tamoxifen resistance in clinical practice. To further substantiate the correlation between aberrant Wnt signalling and acquired Tamoxifen resistant breast cancer, we also investigated the effects of modulating Wnt signalling pathway activity via recombinant Wnt proteins and the Wnt inhibitor, IWP-2 in this model cell line.

## Methods

### Cell culture

The human breast adenocarcinoma cell line MCF7 was obtained from American Type Culture Collection (Manassas, VA, USA), and maintained in Dulbecco’s Modified Eagles Medium (DMEM) (Gibco, Carlsbad, CA, USA). TamR cells were selected from the MCF7 parental cell line grown in graduated concentrations (0.1 μM to 5.0 μM) of 4-hydroxy-Tamoxifen (Sigma Aldrich, Castle Hill, NSW, Australia) over six months. The final concentration of 5 μM was chosen to simulate the pharmacological dosages prescribed to patients, as described previously [23]. TamR cells were maintained in 5 μM of Tamoxifen and DMEM prepared without phenol red indicator. All media contained 5% charcoal stripped foetal bovine serum (Sigma Aldrich), 5% glutamate and 100 units penicillin, 100 μg/mL streptomycin. All cells were grown in a humidified atmosphere of 5% CO_2_, at 37°C and were demonstrated to be free of mycoplasma contamination.

### RNA extraction and cDNA synthesis

RNA was extracted using the RNeasy mini kit (Qiagen, Valencia, CA, USA) following manufacturer’s instructions. Final concentrations were determined using the Nanodrop DA-1000 Spectrophotometer. Only samples with an absorbance of 260/280 nm at a ratio between 2.0 and 2.1 were used for cDNA synthesis. 1 μg of RNA was purified from genomic DNA using DNase I (Invitrogen, Carlsbad, CA, USA) and reverse transcribed to cDNA using the QuantiTect® Reverse Transcription Kit (Qiagen) as per manufacturer’s instructions. To verify that the cDNA synthesized was free of genomic DNA contamination, an additional control reaction devoid of Quantiscript® Reverse Transcriptase was conducted for each purified RNA sample. The resulting cDNA product was then used as a template for PCR amplification.

### Quantitative RT-PCR (qPCR)

A 25 μl qPCR consisting of 25 ng diluted cDNA, QuantiFast® Sybr Green Dye (Qiagen) and 0.1 μM of each qPCR primer pair was performed to obtain quantifiable expressions of Wnt and EMT-related gene targets in MCF7 and TamR cells. All qPCR was conducted in a Stratagene MxPro™3005P. Each sample was repeated in triplicate and normalized against the three housekeeping genes SDHA (Succinate dehydrogenase complex subunit A), HSPCB (Heat shock 90kD protein 1, beta) and YWHAZ (Tyrosine 3-monooxygenase/tryptophan 5-monooxygenase activation protein, zeta polypeptide). The mRNA expressions of the genes of interest were standardized against the geometric mean of the three control genes using the Vandesompele normalisation method [22]. The expression values of TamR cells relative to that of MCF7 cells and expressed as fold-change. All experiments contained a “no amplicon” negative control. Primer sequences were as follows (5^′^ to 3^′^): ER forward (F) CCACCAACCAGTGCACCATT, ER reverse (R) GGTCTTTTCGTATCCCACCTTTC, HER2 F GGGAAGAATGGGGTCGTCAAA, HER2 R CTCCTCCCTGGGGTGTCAAGT, Axin2 F TGTCTTAAAGGTCTTGAGGGTTGAC, Axin2 R CAACAGATCATCCCATCCAACA, CCNDD1 F GGCGGAGGAGAACAAACAGA, CCND1 R TGGCACAAGAGGCAACGA, ROR2 F CGACTGCGAATCCAGGACC, ROR2 RGGCAGAACCCATCCTCGTG, CDH1 F AGGCCAAGCAGCAGTACATT, CDH1 R ATTCACATCCAGCACATCCA, VIM F CCAAACTTTTCCTCCCTGAACC, VIM R GTGATGCTGAGAAGTTTCGTTGA, TWIST1 F GCCAATCAGCCACTGAAAGG, TWIST1 R TGTTCTTATAGTTCCTCTGATTGTTACCA, SDHA F TGGGAACAAGAGGGCATCTG, SDHA R CCACCACTGCGCGGTTCTTG, HSPCB FAAGAGAGCAAGGCAAAGTTTGAG, HSPCB R TGGTCACAATGCAGCAAGGT, YWHZA F ACTTTTGGTACATTGTGGCTTCAA, YWHZA R CCGCCAGGACAAACCAGTAT.

### RT^2^ Profiler PCR array

The Human Wnt Signalling Pathway (PAHS-043, Qiagen) and our own custom designed (CAPH-10800), Qiagen) RT^2^ Profiler PCR Arrays were utilized to investigate a panel of 84 Wnt specific and 84 EMT related genes in TamR and MCF7 cells as per manufacturer’s instructions. Briefly, 500 ng of RNA was converted to cDNA using RT^2^ First Strand Kit (Qiagen). The resultant cDNA product was immediately amplified by qPCR using RT^2^ SYBR Green qPCR Master Mix (Qiagen), using a Stratagene MxPro™3005P. The C_t_ values (threshold cycle) for both cell lines were evaluated using the provided web-based portal (http://pcrdataanalysis.sabiosciences.com/pcr/arrayanalysis.php) and normalised to five housekeeping genes. This analysed data comprised of fold-regulations, which represents the normalized gene expression in the TamR cells compared against the normalized gene expression in the MCF7 cells. Our criteria for a significant differential expression was set at a greater or less than 2.5-fold regulation in TamR cells when compared against MCF7 cells.

### Proliferation assay

Cell proliferation was measured via MTT Cell Proliferation Kit I (Roche, Basel, Switzerland) following the manufacturer’s instructions. Briefly, TamR and MCF7 cells were seeded into a 96-well plate with a concentration of 4000 cells per well, then either left unstimulated or stimulated with 0.1 μg/ml recombinant Wnt3a (rWnt3a, R&D Systems) for 48 hours. Cells were then treated with 5 μM of Tamoxifen (Sigma) or 50% Dimethyl sulfoxide (DMSO) (Sigma) for the final 24 hours. All cells were then labelled with MTT ((3-(4,5-Dimethylthiazol-2-yl)-2,5-diphenyltetrazolium bromide), incubated in a humidified atmosphere of 5% CO_2_, at 37°C for 4 hours and absorbance measured on a microplate reader (SpectraMax M2). The raw absorbance was subsequently measured in 10 replicates at 572 nm, readouts averaged and adjusted accordingly.

All cell numbers were determined using the Countess® Automated Cell Counter (Invitrogen) and further verified via manual cell counting with an aid of a haemocytometer to ensure accurate seeding of cells.

### Wnt reporter assay

MCF7 and TamR cells were plated at a concentration of 5000 cells/well on white bottomed 96 well plates. Cells were serum starved overnight and co-transfected with 0.2 μg of either TOPflash (2 sets of 3 copies (the second set in the reverse orientation) of the TCF binding site) or FOPflash (2 full and one incomplete copy of the TCF binding site (mutated) followed by 3 copies in the reverse orientation) expression plasmids (Millipore, Temecula, CA, USA), and 0.1 μg pRL-TK (Renilla-TK-luciferase vector, Promega) as a control, using Lipofectamine2000. Cells were subsequently treated with recombinant Wnt3a (rWnt3a 0.1 μg/ml) for 48 hours prior to luciferase activities being measured using a Glomax 96 Microplate Luminometer (Turner Biosystems Instrument, Sunnyvale, CA, USA). Firefly luciferase activity was normalized for transfection efficiency by dividing by the Renilla luciferase activity. The TOP/FOP ratio was used as a measure of β-catenin driven transcription. Average activity and standard deviations were derived from octopulate transfected samples.

### Statistical analysis

The data represented in the results section are presented as means with error bars representing standard deviation (SD). The two-tailed unpaired t-test was used to determine the significance. The following symbols were used to denote statistical significance * P < 0.05, ** P < 0.01, *** P <0.001.

## Results

### TamR cells are resistant to tamoxifen

**Figure 1:**
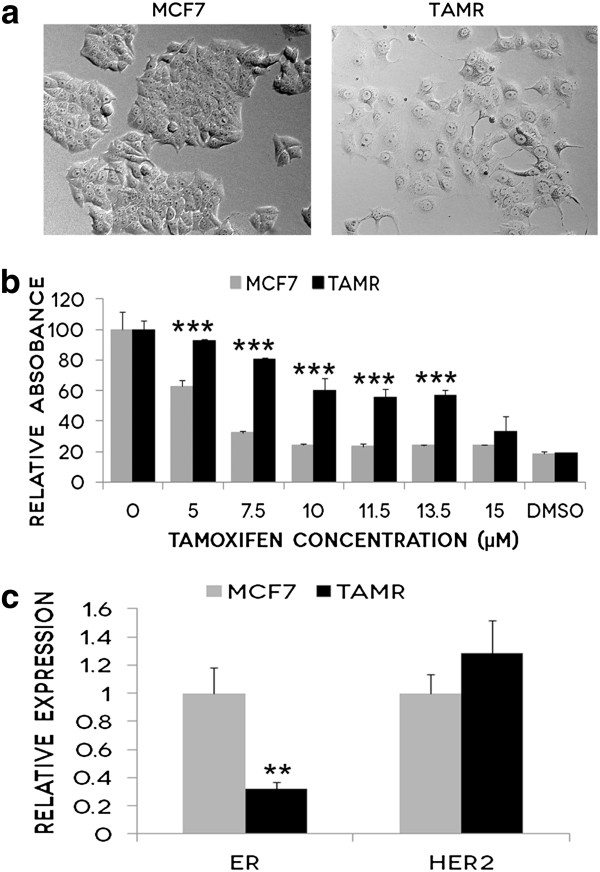
**TamR cells are resistant to Tamoxifen. a**: TamR cells were larger, flatter and exhibited a more mesenchymal phenotype than the parental cell line MCF7. 10X magnification. **b**: MTT proliferation assays were performed on parental MCF7 cells and TamR cells treated with increasing doses of Tamoxifen(0.0–15.0 μM) over 24 hours. 50% DMSO was used as a control as it is known to eliminate cells independent of ER status. TamR cells were successfully selected for acquired Tamoxifen resistance from its parental cell line MCF7 as they continued to proliferate in Tamoxifen concentrations, 5 μM, 7.5 μM, 10 μM, 11.5 μM and 13.5 μM, that eliminate MCF7 cells. Graph represents the average cell proliferation in percentage of 10 replicates with standard deviation represented by error bars. *** P<0.001. Abbreviation: Dimethyl sulfoxide (DMSO). **c**: mRNA expression of ER and HER2 was measured using qPCR. Graph represents the mRNA fold-regulation values of TamR cells relative to MCF7 cells, normalized against three housekeeping genes with standard deviation of triplicate experiments represented by error bars** P<0.01. Abbreviations: Estrogen receptor alpha (ER), Human Epidermal Growth Factor Receptor 2 (HER2).

TamR cells appeared larger in size and flatter, than the parental MCF7 cell line (Figure 1a). They were less likely to form large aggregates of cells, and appeared less adherent than MCF7 cells (Figure 1a). MTT proliferation assays were used to confirm that the laboratory developed model cell line was indeed resistant to the anti-proliferative effects of Tamoxifen (Figure 1b). TamR cells exhibited statistically significant resistance to the Tamoxifen at concentrations ranging from 5 μM to 13.5 μM, well above the clinical dose [23]. Many previous studies have noted that acquired resistance to Tamoxifen is often accompanied by an increased expression of Her2, and a decreased expression of ERα [24,25], which we confirmed in our TamR cells using qPCR (Figure 1c).

### TamR cells exhibit increased Wnt signalling

**Figure 2:**
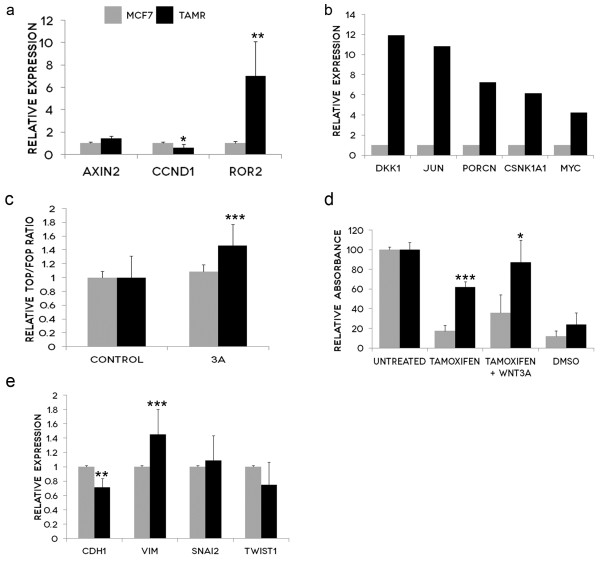
**TamR cells exhibit increased Wnt signalling. a**: mRNA expression of Wnt-related genes was measured using qPCR. Graph represents the mRNA fold-regulation values of TamR cells relative to MCF7 cells, normalized against three housekeeping genes with standard deviation (s.d) of triplicate experiments represented by error bars. *P<0.05, ** P<0.01. **b**: mRNA expression changes of Wnt target genes were measured using RT Profiler PCR arrays and normalised to five housekeeping genes. Criteria for significant change include a statistically significant validation determined by the manufacturer and fold regulation of greater or less than 4. **c**: MCF7 and TamR cells were co-transfected with pRL-TK (Renilla) and either TOPflash or FOPflash expression plasmids. Cells were subsequently treated with 0.1 μg/ml recombinant Wnt3a (rWnt3a) for 24 hours prior to luciferase activities being measured using a Glomax 96 Microplate Luminometer. Average activity and s.ds were derived from octopulate transfected samples. Results represent the average of 3 experiments and bars represent the s.d of the mean. ***P<0.001. **d**: MTT proliferation assays were performed on parental MCF7 cells and TamR cells treated with 5 μM Tamoxifen and 0.1 μg/ml recombinant Wnt3a (rWnt3a) for 24 hours. 50% DMSO was used as a control. Treatment with rWnt3a and 5 μM Tamoxifen inhibited the proliferation of both the MCF7 and TamR cells compared to treatment with Tamoxifen alone. rWnt3a treatment in the TamR cells enhanced their resistance to Tamoxifen back to near basal levels. Graph represents the average cell proliferation in percentage of 10 replicates with s.d represented by error bars. *P<0.05, *** P<0.001. **e**: mRNA expression of EMT markers was measured using qPCR. Graph represents the mRNA fold-regulation values of TamR cells relative to MCF7 cells, normalized against three housekeeping genes with s.d of five independent experiments represented by error bars. **P<0.01, ***P<0.001*.

In addition, TamR cells showed increased expression of the direct Wnt target gene, Axin2 and the non-canonical Wnt receptor Ror2 while exposed to Tamoxifen (Figure 2a). CyclinD1 (CCND1) expression was significantly decreased in TamR cells compared with the parental cell line, MCF7 (Figure 2a). The Wnt RT Profiler PCR array further indentified a number of canonical and non-canonical Wnt genes whose transcription was significantly increased (greater than 4 fold) in the TamR cell line. These were DKK1, JUN, PORCN, CSNK1A1 and MYC (Figure 2b).

This apparent transcriptional upregulation of the canonical (β-catenin dependent) Wnt pathway was confirmed using the the well established TOPflash/FOPflash Wnt reporter assay. Stimulation with human recombinant Wnt3a (rWnt3a) had no effect on MCF7 cells, but significantly increased the transcription of the Wnt luciferase reporter in the TamR cells (Figure 2c). Next, we tested the effect of rWnt3a treatment on cell proliferation and Tamoxifen resistance. Treatment with rWnt3a and 5 μM Tamoxifen inhibited the proliferation of both the MCF7 and TamR cells compared to treatment with Tamoxifen alone. Furthermore, rWnt3a treatment in the TamR cells enhanced their resistance to Tamoxifen back to near basal levels (Figure 2d).

### TamR cells have increased expression of EMT markers

Because the TamR cells displayed some morphological differences from the parental MCF7 cells, as well as the emerging links between Wnt signalling and EMT, we conducted further expression profiling which confirmed TamR cells express some of the hallmarks of EMT. Compared to their parental cell line MCF7, TamR cells exhibited significantly decreased expression of E-cadherin (CDH1) and significantly increased expression of Vimentin (VIM) (Figure 2e). In addition, Slug (SNAI2) showed a modest non significant increase in expression, and Twist (TWIST1) showed a slight decrease in expression.

### The Wnt inhibitor, IWP-2, inhibits cell proliferation and EMT

**Figure 3:**
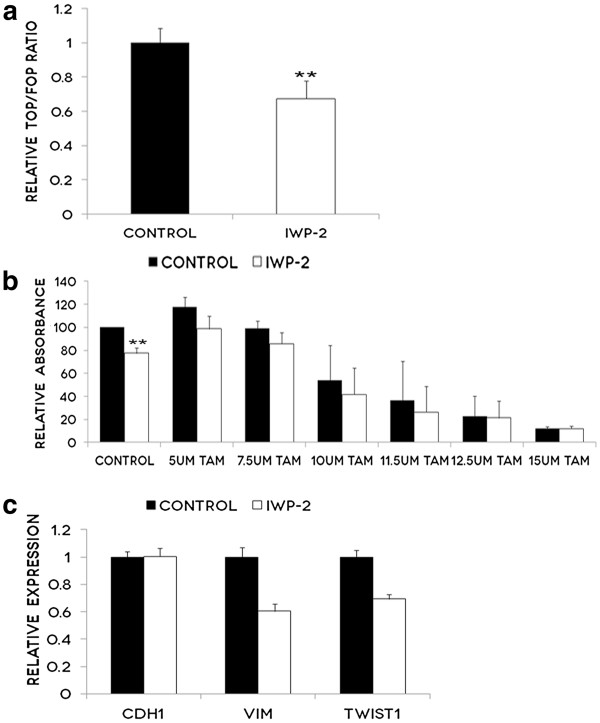
**The Wnt inhibitor IWP-2 inhibits proliferation and EMT in TamR cells. a**: TamR cells were pre-treated with 5 μM IWP-2 for 24 hours prior to co-transfection with pRL-TK (Renilla) and either TOPflash or FOPflash expression plasmids. Cells were subsequently treated with 5 μM IWP-2 for 24 hours prior to luciferase activities being measured using a Glomax 96 Microplate Luminometer. Average activity and standard deviations were derived from triplicate transfected samples. Results represent the average of 4 experiments and bars represent the standard deviation (s.d) of the mean. **P<0.01. **b**: MTT proliferation assays were performed on TamR cells treated with increasing doses of Tamoxifen(0.0–15.0 μM) over 24 hours, with or without the addition of 5 μM IWP-2. The graph represents the average cell proliferation of triplicate wells in three independent experiments, with standard deviation represented by error bars. ** P<0.01. **c**: mRNA expression of EMT markers was measured using qPCR in TamR cells treated for 48 hours with 5 μM IWP-2. Graph represents the mRNA fold-regulation values of control cells relative to IWP-2 treated cells, normalized against three housekeeping genes. This is a single experiment, and error bars represent the standard deviation of triplicate wells.

We first confirmed that the Wnt inhbitor, IWP-2, was effective in our cell line by treating TamR cells with 5 μM IWP-2 and determining the effects on TCF/LEF mediated transcription, as measured by a TOPflash/FOPflash Wnt reporter assay. IWP-2 significantly inhibited Wnt signalling in the TamR cell line (Figure 3a). To determine if inhibition of Wnt signalling would alter the proliferation of TamR cells and sensitivity to Tamoxifen, we treated cells with increasing concentrations of Tamoxifen in the presence or absence of IWP-2. Treatment with IWP-2 alone significantly reduced proliferation by approximately 20% (Figure 3b). At Tamoxifen concentrations of 5–11.5 μM, the addition of IWP-2 further inhibited cell proliferation (though this was not statistically significant) providing some support for future research into combination therapy targeting ER and Wnt signalling (Figure 3b). In addition, IWP-2 treatment resulted in decreased expression of the key EMT transcription factors, Vimentin and Twist (Figure 3b).

## Discussion

Despite the development of aromatase inhibitors and therapies targeting other key proteins involved in breast carcinogenesis, Tamoxifen remains in widespread clinical use. However, both *de novo* and acquired resistance to Tamoxifen occur frequently in the clinical management of breast cancer patients. Understanding the mechanisms behind resistance will be important for not only improving treatment success, but in understanding the key signalling pathways involved in breast carcinogenesis.

At present, research has considered relatively few signalling pathways and translation of these works to the clinical setting has proved to be insufficient in restoring Tamoxifen sensitivity [26]. There is an imperative need to identify previously unconsidered mechanisms for successful modulation of therapeutic response in this aggressive subtype of breast cancer.

In this study, we considered the Wnt signalling pathway as a potential mechanism involved in acquired Tamoxifen resistant breast cancer. This was based on substantial evidence in the literature implicating aberrant activation of Wnt signalling in aggressive breast tumour subtypes including triple negative breast cancers, which are known to exhibit *de novo* resistance to Tamoxifen [7,9,10]. Here, we have extended this association to include acquired Tamoxifen resistant cells by providing data that largely indicate increased activation of both canonical and non-canonical Wnt pathways in our TamR cells.

AXIN2, MYC, DKK1 and CCND1 are well-documented downstream canonical Wnt-target genes that regulate cell proliferation, metastasis and tumourigenesis [27-30]. Our data, which revealed a upregulation of AXIN2, DKK1 and MYC in the TamR cells, are largely consistent with findings documented in current literature on triple negative breast cancer [7,31-36]. In particular, a review by Bouchalova et al. (2009) classified MYC amplifications as the most frequent aberrations in triple negative breast cancer [32]. Additionally, AXIN2, the most reliable endogenous gene target of Wnt canonical pathway [28] activation was markedly upregulated upon mRNA analysis of high-grade breast tumours [7,27]. Our study showed a trend to increased expression of AXIN2, though this was not statistically significant.

Interestingly, our findings for CCND1 showed a two fold downregulation in the TamR cells compared to that of MCF7 cells. This differs from our findings on other Wnt target genes as well as some evidence in current literature. Elevated expression of CCND1 was commonly associated with a more aggressive breast disease phenotype and an adverse patient outcome [37]. A reason for this discrepancy could be due to the lack of specificity of CCND1 as a downstream Wnt signalling gene target [28,38]. It has been shown that a wide variety of mitogenic signalling pathways in addition to the Wnt pathway converge at the level of CCND1 mRNA and/or protein up-regulation [38]. Furthermore, a number of studies have disputed whether CCND1 is indeed a canonical Wnt target gene at all [39,40]. Despite this, our combined results of the three up-regulated Wnt gene targets and increased Wnt reporter gene activity are indicative of canonical Wnt pathway activation in TamR cells. However, while DKK1 is identified as a downstream target of canonical Wnt signalling, this is suspected to be as part of a negative feedback loop as DKK1 is an inhibitor of the canonical co-receptor LRP [29]. Therefore it is possible that the upregulation of the canonical Wnt pathway is tempered somewhat by the increased expression of DKK1. The increase in expression of CK1alpha (CSNK1A1), a critical regulator of the APC destruction complex [41] supports the activation of the canonical Wnt pathway in response to Tamoxifen [42]. Furthermore, stimulation of TamR cells with rWnt3a protein potentiated Tamoxifen resistance in TamR cells. Collectively, this data adds to the increasing body of evidence implicating the increased activation of canonical pathway with aggressive subtypes of breast cancer.

A strong increase in expression of Porcupine (PORCN) was also noted, which is essential for secretion of all Wnt ligands from the endoplasmic reticulum. This suggested that the increase in Wnt signalling may be network-wide and not specific to a particular arm of the pathway. Interestingly genes involved in the β-catenin independent or non-canonical Wnt pathways appear to also be upregulated following acquired Tamoxifen resistance. Expression of both the downstream non-canonical Wnt target gene JUN and the recently identified Wnt5a receptor, ROR2 were also increased in TamR cells. These data suggest an upregulation of the entire Wnt signalling network which should be further explored, particularly in light of recent studies identifying the importance of ROR2 driven signalling in human cancer [43]. The addition of the Wnt inhibitor, IWP-2 which should target both arms of the Wnt pathway, appeared to further enhance the anti-proliferative effects of Tamoxifen in this model of acquired Tamoxifen resistance.

TamR cells also exhibited altered transcription of a number of key genes involved in EMT. This shift to a more mesenchymal phenotype fits with the current literature suggesting that drug treatment can effect cellular behaviour and plasticity, with EMT linked to chemoresistance in a number of cell lines and tumour types, including breast cancer [22,44-46].

## Conclusions

In conclusion, this study provides insight into the role of Wnt signalling pathway in acquired Tamoxifen resistant breast cancer. Combined, our data suggest that acquisition of resistance to Tamoxifen is accompanied by an increase in the Wnt signalling pathway and a transition to a more mesenchymal phenotype. Future research should therefore consider members of this pathway as one of the potential targets to be used in combination therapy for successful restoration of acquired Tamoxifen sensitivity. This raises the possibility that drugs targeting the Wnt signalling pathway, many of which are already in development, could be added to the arsenal of drugs for individualised and targeted treatment in breast cancer.

## Abbreviations

AXIN2: Axis Inhibition Protein 2; EMT: Epithelial to mesenchymal transition; ER: Estrogen receptor alpha; HER2: Human Epidermal Growth Factor Receptor 2 (HER2); HSPCB: Heat shock 90kD protein 1, beta; qPCR: Quantitative reverse transcriptase PCR; SERM: Selective Estrogen Receptor Modulator; SDHA: Succinate dehydrogenase complex subunit A; TamR: Tamoxifen Resistant cell line; YWHAZ: Tyrosine 3-monoox-ygenase/tryptophan 5-monoox-ygenase activation protein, zeta polypeptide

## Competing interests

The authors declare that they have no competing interests.

## Authors’ contributions

YL, EH, LB, EJ and CF performed the experiments. CF conceived the study and drafted the manuscript. RW participated in the design of the study, and helped draft the manuscript. All authors read and approved the final manuscript.

## Pre-publication history

The pre-publication history for this paper can be accessed here:

http://www.biomedcentral.com/1471-2407/13/174/prepub
